# Prevalence of bacterial vaginosis in pregnancy in a tertiary health institution, south western Nigeria

**DOI:** 10.11604/pamj.2019.33.9.17926

**Published:** 2019-05-07

**Authors:** Olusola Peter Aduloju, Akinyemi Akinsoji Akintayo, Tolulope Aduloju

**Affiliations:** 1 Department of Obstetrics and Gynaecology, Ekiti State University, Ado-Ekiti, Nigeria; 2 Medical Social Services Department, Ekiti State University Teaching Hospital Ado-Ekiti, Nigeria

**Keywords:** Prevalence, abnormal vaginal discharge, bacterial vaginosis, pregnancy, south western Nigeria

## Abstract

**Introduction:**

Bacterial vaginosis (BV) is the most common cause of vaginal discharge in women of child bearing age. Bacterial vaginosis has emerged as a global health issue due to the adverse outcome in pregnancy and in the puerperium. The study determined the prevalence of BV and outcome of delivery among pregnant women.

**Methods:**

Socio-demographic data and vaginal swab samples were obtained from 362 consecutive pregnant women with abnormal vaginal discharge attending antenatal clinic in Ekiti State University Teaching Hospital, Ado-Ekiti. Data were analysed using SPSS statistical software 21 and association between variables was compared using Chi square.

**Results:**

The prevalence of BV among pregnant women with abnormal vaginal discharge in this study was 16.6%. Age group 25-34yrs, multiparity and higher education were significantly associated with BV, p < 0.05. Symptoms such as vulvar itching, dyspareunia, lower abdominal pains and characteristic of vaginal discharge such as colour and consistency were significantly associated with BV, p < 0.05. Women with bacterial vaginosis significantly had prelabour rupture of fetal membrane and their babies were born prematurely with low birth weight and Apgar score of less than 5 at one minute, p < 0.05. However, there was no difference statistically in rate of admission into special care baby unit among the women, p > 0.05.

**Conclusion:**

The findings of this study suggest that there should be screening for BV in pregnant women presenting with abnormal vaginal discharge so that they could be treated accordingly. This will mitigate the complications arising from bacterial vaginosis.

## Introduction

In healthy women, the vaginal environment is a balanced ecosystem characterized by the presence of various species of Lactobacillus. These bacteria inhibit the growth of other microorganisms through various mechanisms including production of organic acids such as lactic acid and other antimicrobial substances (hydrogen peroxide and bacteriocins), competition for mannose and glycoprotein receptors and adhesion to the epithelium. The depletion of these vaginal Lactobacilli is known to be associated with bacterial vaginosis [1, 2]. Bacterial vaginosis (BV) is a poly-microbial syndrome characterized by the loss of normal vaginal flora, predominantly hydrogen peroxide producing Lactobacillus spp., and the increase in the number and species of other bacteria in vaginal fluid. The decrease in lactobacilli and increase in facultative and anaerobic bacteria may lead to changes in the characteristics of vaginal fluid such as thickness and odour of the discharge [3, 4]. The prevalence of bacterial vaginosis among non-pregnant women ranges from 15-30% [5-8] and in pregnancy, it ranges between 11-16% in developed countries [6, 7], 21-29% in Kenya and South Africa [9, 10] while in Nigeria, a prevalence rate of 17%, 17.3% and 25% have been reported from separate studies done in the south-east, north-east and south-west Nigeria respectively [11-13]. Bacterial vaginosis are associated with numerous health problems. It has been related to many gynaecological conditions including pelvic inflammatory disease, post hysterectomy vaginal cuff cellulitis, endometritis and post-abortal sepsis [5]. In pregnancy, BV has been associated with poor pregnancy outcomes. It increases the risk of miscarriage, preterm labor, preterm delivery, chorioamnionitis and postpartum complications such as endometritis and wound infections [4, 11]. Pregnancy is commonly associated with increased vaginal discharge which is often non-pathological while bacterial vaginosis, candidiasis and trichomoniasis are associated with abnormal vaginal discharge [12]. However, most cases of abnormal vaginal discharge in pregnant women are treated inappropriately as candidiasis as a result of inadequate investigation for other causes. The diagnosis of BV can be made using Amsel´s clinical criteria [14] or in the laboratory by scoring bacterial morphotypes from a Gram stain of vaginal fluid [15]. This study examined vaginal discharge from pregnant women attending the prenatal booking clinic of the Ekiti State University Teaching Hospital (EKSUTH), Ado-Ekiti, south-western Nigeria to determine the prevalence of bacterial vaginosis and outcomes of delivery among the women.

## Methods

The study was a descriptive cross-sectional study conducted in the Department of Obstetrics and Gynaecology, EKSUTH, Ado-Ekiti, south-western Nigeria. The inclusion criteria were pregnant women who presented in the antenatal clinic with abnormal vaginal discharge and who consented to participate in the study while patients who douched with chemicals, those who had used antibiotics in the preceding 4 weeks and those who did not consent were excluded from the study. Consecutive pregnant women with abnormal vagina discharge, who met the inclusion criteria and had been adequately counseled were recruited into the study from April 2017 to June 2017. They were followed up to the point of delivery especially those that had BV.

**Sample size determination:** a sample size of 362 women was determined using the single proportion sample size determination formula and a prevalence rate 31.5% of abnormal vaginal discharge in pregnancy from a previous study [12]. The degree of precision was 5% with a 95% confidence interval and 10% attrition rate for non-respondents.

**Data collection:** data was collected from the women with the use of coded questionnaires which were in three sections. The first section enquired about socio-demographic variables like age, marital status, educational status, occupation of woman and husband and parity; the second section elicited about the gestational age at which test was done and at delivery, antenatal complications and outcome of delivery such as birth weight, Apgar scores at birth and admission into special care baby unit (SCBU) while the third section obtained the results of the various criteria for the diagnosis of BV.

**Specimen collection:** each pregnant woman was placed in dorsal position and had general and urogenital examination conducted for abnormalities such as erythema, excoriation marks and discharge. A sterile disposable Cusco's speculum was passed and specimens were collected using a sterile cotton swab- with an identification mark which tallied with the coded questionnaire- from lateral and posterior fornices of the vagina. The swab was smeared on grease-free slide which had been marked with number of the corresponding questionnaire of the participant to air dry and the colour of the specimen was noted. The pH of the specimen was determined using the pH indicator paper and the cotton swab was dipped inside a glass tube containing one milliliter of 10% potassium hydroxide (10% KOH) and sniffed for the characteristic fishy odour. All the findings were immediately recorded on the coded questionnaire for each participant.

**Microscopy/wet preparation:** the specimens on the marked grease-free slides of the participants were examined under compound light microscope for presence of clue cells. A gram stained smear of each specimen was counter-stained with safranin and evaluated for BV by Spiegel's method [16].

**Clinical diagnosis of BV:** BV was diagnosed using any three of the four criteria recommended by Amsel [14]. These criteria are: presence of homogenous white-grey vaginal discharge; presence of clue cells (>20% of epithelial cells with clue cells) on wet mount; a fishy amine odour of the vaginal discharge before and after addition of 10% KOH (positive whiff test) and a vaginal pH of > 4.5.

**Gram staining diagnosis of BV:** each slide was then graded as per the standardized quantitative morphological classification method developed by Nugent *et al*. [15] which assigns a score between 0 and 10 based on the following bacterial morphotypes: large gram-positive rods (*Lactobacillus* morphotypes), small gram-variable rods (*G. vaginalis* morphotypes), small gram-negative rods (*Bacteroides* spp. morphotypes), curved gram-variable rods (Mobiluncus spp. morphotypes), and gram-positive cocci. Each morphotype was quantitated from 1 to 4+ with regards to the number of morphotypes per oil immersion field (0, no morphotypes; 1+, less than 1 morphotype; 2+, 1 to 4 morphotypes; 3+, 5 to 30 morphotypes; and 4+, 30 or more morphotypes). Scores between 0 and 3 represented “normal vaginal flora,” scores between 4 and 6 represented “intermediate vaginal flora,” and scores between 7 and 10 were considered diagnostic for bacterial vaginosis.

**Data analysis:** data collected were analyzed using the IBM Statistical Package for Social Sciences version 20 (SPSS Chicago, IL, USA). Categorical variables were presented in frequency and percentages with test of significance done using Chi square while continuous variables were expressed in mean and standard deviation with significance test done using student t test. Level of significance was set at p value < 0.05.

**Ethical consideration:** the study was explained to the participants and informed verbal and written consent was obtained from them. The patients were at liberty to withdraw from the study without affecting their treatment. Information obtained from the participants will be kept strictly confidential. Ethical clearance for the study was obtained from the Ethics and Research Committee of Ekiti State University Teaching Hospital, Ado-Ekiti.

## Results

A total of 362 pregnant women who had abnormal vaginal discharge during the study period were recruited. Out of these women with abnormal vaginal discharge, 60 women had bacterial vaginosis giving a prevalence rate of 16.6%. Fifty seven (95.0%) of these women were positive for bacterial vaginosis using both the clinical criteria and gram stain morphology, 2 (3.3%) were positive using the clinical criteria alone while 1 (1.7%) women had bacterial vaginosis using the gram stain morphology only. The overall accuracy of the clinical criteria and the gram stain morphology in the diagnosis of bacterial vaginosis in this study is 95%. Table 1 showed that there was no significant difference in the mean age, parity, gestational age at which test was done and gestational age at delivery among the women with or without bacterial vaginosis; p > 0.05. Table 2 showed that the age group, parity, education, and marital status of the women as well as trimester of pregnancy when test was done showed significant difference between the women in the two groups, p < 0.05 while the other socio-demographic characteristics were also shown in the table. The two groups of women showed significant difference in the colour and consistency of the vaginal discharge and some other symptoms such as dysuria, dyspareunia and lower abdominal pains; p < 0.05 while other symptoms were not significantly different in them; p > 0.05 as shown in Table 3. Table 4 showed that more women with bacterial vaginosis had prelabour rupture of fetal membrane and had babies with prematurity, low birth weight and Apgar score less than 5 at one minute than women without bacterial vaginosis and these were statistically significant, p < 0.05.

**Table 1:** Obstetric characteristics of the pregnant women involved

Characteristics	Frequency of vaginal discharge		
	Bacterial vaginosis present (Mean ± SD)	Bacterial vaginosis absent (Mean ± SD)	P value
Age of women (years)	26.24 ± 6.14	28.13 ± 4.38	0.459
Parity	2.73 ± 2.33	3.02 ± 1.12	0.074
Gestation at test(weeks)	25.27 ± 3.42	26.63 ± 2.52	0.524
Gestation at delivery (weeks)	36.56 ± 7.24	37.13 ± 1.78	0.825

SD: Standard deviation

**Table 2:** Sociodemographic characteristics of the pregnant women involved

Characteristics	Frequency of vaginal discharge		
	Bacterial vaginosis present (n = 60)	Bacterial vaginosis absent (n = 302)	P value
**Age of women (years)**			
≤ 19	1 (1.6%)	6 (2.0%)	0.035*
20-24	10 (16.7%)	68 (22.5%)	
25-29	24 (40.0%)	121 (40.1%)	
30-34	16 (26.7%)	59 (19.5%)	
35-39	6 (10.0%)	38 (12.6%)	
≥ 40	3 (5.0%)	10 (3.3%)	
**Parity**			
0	0 (15.0%)	39(12.9%)	0.045*
1-4	45 (75.0%)	244 (80.8%)	
≥ 5	6 (10.0%)	19 (6.3%)	
**Education of women**			
Primary	9 (15.0%)	39 (12.9%)	0.002*
Secondary	19 (31.7%)	98 (32.5%)	
Tertiary	32 (53.3%)	162 (53.6%)	
**Occupation of women**			
Unemployed	8 (13.3%)	40 (13.2%)	0.072
Self-employed	13 (21.7%)	79 (26.2%)	
Privately employed	10 (16.7%)	53 (17.5%)	
Government employed	29 (48.3%)	130 (43.1%)	
**Marital status**			
Single	3 (5.0%)	7 (2.3%)	0.018*
Married	57 (95.0%)	295 (97.7%)	
**Lifetime sex partners**			
1	13 (21.7%)	173 (57.3%)	0.021*
≥ 2	48 (79.3%)	129 (42.7%)	
**Trimester of pregnancy**			
1^st^ trimester	2 (3.3%)	10 (3.3%)	0.001*
2^nd^ trimester	43 (71.7%)	133 (44.1%)	
3^rd^ trimester	15 (25.0)	159 (52.6%)	

* Statistically significant

**Table 3:** Symptomatology of the pregnant women with bacterial vaginosis

Symptoms†	Bacterial vaginosis present n= 60 (%)	Bacterial vaginosis absent n= 302 (%)	P value
Vulva itching	36 (18.0%)	164 (82.0%)	0.075
Dysuria	49 (56.9%)	37 (43.1%)	0.047*
Dyspareunia	41 (68.3%)	19 (31.7%)	0.035*
Lower abdominal pains	35 (62.5%)	21 (37.5%)	0.025*
**Colour of discharge**			
White	4 (2.2%)	176 (97.8%)	0.001*
Yellow	52 (53.1%)	46 (46.9%)	0.019*
Grey	4 (4.8%)	80 (95.2%)	0.025*
Consistency of discharge			
Thick	8 (3.7%)	207 (96.3%)	0.039*
Watery	49 (67.1%)	24 (32.9%)	0.001*
Frothy	3 (4.1%)	71 (95.9%)	0.018*
**Odour of discharge**			
Normal	9 (21.4%)	33 (78.6%)	0.452
Malodorous	51 (19.6%)	209 (80.4%)	0.724

* Statistically significant

† Some women presented with more than one symptom

**Table 4:** Obstetric outcome of the women involved in the study

Outcome/complication	Bacterial vaginosis present n= 60 (%)	Bacterial vaginosis absent n= 302 (%)	P value
PROM	14 (63.6%)	8 (36.4%)	0.015*
Low birth weight	10 (76.9%)	3 (23.1%)	0.035*
Prematurity	15 (71.4%)	6 (28.6%)	0.047*
†Birth asphyxia	5 (33.3%)	10 (66.7%)	0.001*
‡SCBU admission	7 (46.7%)	8 (53.3%)	0.352
No complication	9 (3.3%)	267 (96.7%)	0.001*

* Statistically significant

PROM – Premature rupture of membranes

† Birth Asphyxia - Apgar score < 5

‡ SCBU - Special care baby unit

## Discussion

The prevalence of 16.6% recorded in this study lies within the documented reported prevalence of 6%-32% from studies conducted in different regions of the world [17]. This prevalence is also consistent with figures reported from south-east and north-east Nigeria by Adinma *et al.* [11] and Ibrahim *et al.* [12] but higher than finding of Larrson *et al.* [18] and lower than findings of Afolabi *et al.* [19] and Isik *et al.* [20]. Higher prevalence rates of bacterial vaginosis than in the present study were also reported from other sub-Saharan countries such as Kenya, Botswana, and Zimbabwe [21]. The variation in the prevalence rates of bacterial vaginosis have been attributed to sociodemographic characteristics, sexual activity, reproductive health information, and behavioral and genital hygiene. The clinical criteria for bacterial vaginosis based on the composite criteria of Amsel *et al.* [14] are used by most researchers on bacterial vaginosis. However, these clinical diagnostic criteria have shown good correlation with the gram staining of vaginal discharge for bacterial vaginosis in subsequent studies [22]. There was an overall agreement of 95% between these two criteria in this study and this is consistent with findings from previous studies [19, 22]. Women in the age group between 25-34years had the highest prevalence of bacterial vaginosis and this was similarly reported in a study by Asiegbu *et al*. [17] but this differs from the age groups reported from studies by Adinma *et al*. [11], Ibrahim *et al*. [12] and Nwadioha *et al*. [23]. However, this study has further revealed that in consistent with previous studies that the highest prevalence of bacterial vaginosis occurred in women within the reproductive age. These women are also the most sexually active and studies have shown that they have the highest rate of pregnancies and sexually transmitted diseases [12, 24]. Women who were multiparous and with higher education constituted the highest group with bacterial vaginosis in this study and this is consistent with reports from previous studies [12, 21, 23, 24]. This was attributed to increased coital frequency and douching which might result in reduction in the physiological barrier in the vaginal and overgrowth of normal commensals [25]. This is in contrast to reports from other studies that revealed low prevalence of bacterial vaginosis among women with higher education. They opined that this higher level of education could be associated with higher level of enlightenment, sophistication and utilization of orthodox medicine by them while those who lack western education patronize traditional medicine more. This high patronage of traditional medicine, which involves insertions into the vagina, predisposes them to having vaginal discharge [12, 25]. This study revealed that women in marital relationship and lifetime sex partners have increased prevalence of bacterial vaginosis but these were not statistically significant. The role of sexual activity in the acquisition of bacterial vaginosis is not clear. Sexual behavior-related characteristics, including number of lifetime male sex partners, multiple male sex partners, and a recent history of new sex partners, have been consistently associated with bacterial vaginosis. Studies have shown that multiple or new sex partners increased the risk of acquiring bacterial vaginosis by a factor of 1.6-2.5 and these studies also suggested that condom use may be protective [21, 26]. Prelabour rupture of fetal membrane, preterm delivery and low birth weight were significantly found among women who had bacterial vaginosis in our study. This is consistent with findings of previous studies which have linked BV with low birth weight, preterm birth, PROM, preterm labour, chorioamnionitis, post-caesarean or post-partum endometritis [11, 12, 27, 28]. BV was found to be more frequently associated with preterm than term pregnancy, which is similar to a study carried out in the South Eastern Nigeria [12]. The researchers then suggested the need for adequate screening of pregnant women with vaginal discharge in order to diagnose and treat BV so as to prevent preterm delivery and complications that may result from it. However, Adesiji *et al* [13] did not find any significant association between bacterial vaginosis and low birth weight or preterm birth in their study. They argued that this finding was accurate since women with medical conditions or obstetric complications that could influence the pregnancy outcomes were excluded from the study. The mechanism by which bacterial vaginosis causes the preterm birth of an infant with low birth weight is not known, but there is evidence that it causes infection of the upper genital tract, which in turn causes premature birth. In other studies, bacterial vaginosis has been associated with increased infection of amniotic fluid, infection of the chorion and amnion, and histologic chorioamnionitis. Pregnant women with bacterial vaginosis have elevated vaginal or cervical levels of endotoxin, mucinase, sialidase, and interleukin-1a suggesting that microorganisms that cause bacterial vaginosis stimulate the production of cytokines [29].

## Conclusion

This study showed that bacterial vaginosis do occur among pregnant women with its attendant adverse pregnancy outcomes. All other findings raise the need for health educational programs through different media to educate pregnant women about the difference between normal and abnormal vaginal discharge and when to consult their doctor. There should also be regular screening for bacterial vaginosis during the routine antenatal clinic visits by the health workers to enable early detection and treatment. Further multicentre studies on the pattern of complications of BV in pregnant women with abnormal vaginal discharge are needed to determine future strategies for prevention and treatment of bacterial vaginosis in pregnancy.

### What is known about this topic

- Bacterial vaginosis is the most common cause of vaginal discharge in women of child bearing age;
- Bacterial vaginosis has emerged as a global health issue due to the adverse outcomes in pregnancy and in the puerperium;
- Bacterial vaginosis is a poly-microbial syndrome characterized by the loss of normal vaginal flora, predominantly, predominantly hydrogen peroxide producing Lactobacillus spp., and the increase in the number and species of other bacteria in vaginal fluid.

### What this study adds

- Bacterial vaginosis is still prevalent among the pregnant women in our environment;
- Multiparity, higher education, marital relationship and increased lifetime sexual partners were risk factors associated with bacterial vaginosis;
- There is a need for regular screening for bacterial vaginosis among pregnant women with abnormal vaginal discharge.

## Competing interests

The authors declare no competing interests.
